# One step forward, two steps back: Tensions between malaria elimination and improved malaria surveillance in the Solomon Islands

**DOI:** 10.3934/publichealth.2020067

**Published:** 2020-11-23

**Authors:** Sebastian Kevany

**Affiliations:** University of California, 550 16th Street, San Francisco, California 94158, USA

**Keywords:** malaria, surveillance, health systems, eradication

## Abstract

The Solomon Islands experienced, between 2010, an apparent meteoric fall in the level of malaria incidence and prevalence [1]. Thanks ostensibly to the efforts of bilateral and multilateral partners and donors, annual parasite incidence (API) fell from 70 to 40 per 1,000 population. With such dramatic progress, international efforts were hailed as dramatic successes and showcased as progress towards malaria elimination and eradication, Yet, paradoxically, the true caseload of malaria in the Solomon Islands has revealed a situation that calls for more, rather than less, support.

The Solomon Islands experienced, between 2010, a meteoric fall in the level of malaria incidence and prevalence [1]. Thanks ostensibly to the efforts of bilateral and multilateral partners and donors, annual parasite incidence (API) fell from 70 to 40 per 1,000 population. With such dramatic progress, international efforts were hailed as dramatic successes [2] and showcased as progress towards the *beau ideal:* malaria elimination and eradication, within most of our lifetimes [3].

Yet, in 2017, it appeared that disaster had struck: API caseloads rose, again dramatically, to above 80 per 1,000 population. Explanations as diverse as program failures, globalization, migration, and climate change were cited for this reversal of progress [4], and these were at least in part fair explanations. Ironically, however, the true cause lay in the advancement of the country's heath system: with greater surveillance, resources, skills, and monitoring and evaluation capacity the Solomon Islands was, for the first time, able to provide a true picture of actual malaria prevalence—something that, it transpired, previous estimates had only touched upon.

Although pre-2017 surveillance systems were recognized as being based on incomplete information [5], no one could have been aware of the vast scale of undetected malaria in the county—of the shadow line of actual as opposed to perceived incidence and prevalence, particularly in remote and previously inaccessible areas such as the Malaita and Central provinces, which alone account for the vast majority of reported cases [6].

The true levels of malaria did, however, cause problems for international program support dependent on the demonstration of tangible progress towards both meeting treatment targets and lowering disease levels. In a cruel twist, the country's health system progress had demonstrated that both eradication progress and grant performance was severely underperforming.

This, of course, was not the case: although the relative or perceived success of the program had changed dramatically, the absolute number of those treated for malaria and accessing bed nets had increased dramatically [7]. It is, in retrospect, only through these figures that one can gain a true understanding of the efforts and successes of the National Vector Borne Disease Control Programme.

Yet even with that recognition, the question remains as to whether, based on a limited understanding of the true malaria disease burden, that malaria elimination should have been considered possible [8]. No doubt, such enticing goals are attractive to funders, and excite and invigorate the global health community in to working towards and contributing to individual and collective historical milestones. Yet, too often, such ambitious goals are dispiritingly unmet, leading to the risk of prospective aid scepticism.

More immediately, the Solomon Islands is now subject to a malaria elimination reporting system, requiring a level of effort and detail totally impractical for facility staff in high prevalence provinces. Similar questions might need be faced by donors: are they basing projections and targets, too often, on incomplete information sets?

If so, should greater leniency be shown towards those countries, districts or regions for which health surveillance information is known to be incomplete or unreliable? In turn, do such knowledge limitations question the very assumptions of accurate knowledge on which productive global health programs are based? In a final paradox, the true caseload of malaria in the Solomon Islands has revealed a situation that calls for more, rather than less, support.

## References

[1] Cotter C, Sturrock HJ, Hsiang MS (2013). The changing epidemiology of malaria elimination: new strategies for new challenges. Lancet.

[2] Murray CJ, Ortblad KF, Guinovart C (2014). Global, regional, and national incidence and mortality for HIV, tuberculosis, and malaria during 1990–2013: a systematic analysis for the Global Burden of Disease Study 2013. Lancet.

[3] Feachem RG, Phillips AA, Hwang J (2010). Shrinking the malaria map: progress and prospects. Lancet.

[4] Cohen JM, Smith DL, Cotter C (2012). Malaria resurgence: a systematic review and assessment of its causes. Malar J.

[5] Smith J, Tahani L, Bobogare A (2017). Malaria early warning tool: linking inter-annual climate and malaria variability in northern Guadalcanal, Solomon Islands. Malar J.

[6] Otter M (2002). Development planning for a divided society in a failed state: The case of Solomon Islands. Dev Bull.

[7] Atkinson JA, Bobogare A, Fitzgerald L (2009). A qualitative study on the acceptability and preference of three types of long-lasting insecticide-treated bed nets in Solomon Islands: implications for malaria elimination. Malar J.

[8] Atkinson JA, Johnson ML, Wijesinghe R (2012). Operational research to inform a sub-national surveillance intervention for malaria elimination in Solomon Islands. Malar J.

